# Key Feature Extraction Method of Electroencephalogram Signal by Independent Component Analysis for Athlete Selection and Training

**DOI:** 10.1155/2022/6752067

**Published:** 2022-04-15

**Authors:** Zhongwei Huang, Lifen Cheng, Yang Liu

**Affiliations:** ^1^School of Physical Education, Jiamusi University, Jiamusi 154000, China; ^2^School of Physical Education, Nanchang Normal University, Nanchang 330032, China

## Abstract

Emotion is an important expression generated by human beings to external stimuli in the process of interaction with the external environment. It affects all aspects of our lives all the time. Accurate identification of human emotional states and further application in artificial intelligence can better improve and assist human life. Therefore, the research on emotion recognition has attracted the attention of many scholars in the field of artificial intelligence in recent years. Brain electrical signal conversion becomes critical, and it needs a brain electrical signal processing method to extract the effective signal to realize the human-computer interaction However, nonstationary nonlinear characteristics of EEG signals bring great challenge in characteristic signal extraction. At present, although there are many feature extraction methods, none of them can reflect the global feature of the signal. The following solutions are used to solve the above problems: (1) this paper proposed an ICA and sample entropy algorithm-based framework for feature extraction of EEG signals, which has not been applied for EEG and (2) simulation signals were used to verify the feasibility of this method, and experiments were carried out on two real-world data sets, to show the advantages of the new algorithm in feature extraction of EEG signals.

## 1. Introduction

In over the next two hundred years, the human exploration of the brain will never stop. Study on the brain structure and its related research appeared in particular cognitive function for nearly 10 years, with high performance due to the rapid development of computer technology and to the widespread use of noninvasive brain imaging technology, to become one of the difficult and frontier hot academic research directions. However, understanding and recognizing the neural mechanism behind the mysterious brain is still a basic and difficult problem in current brain science research [1–3]. Among these complicated and challenging problems in brain science, brain plasticity is one of the most important issues. Research on brain plasticity usually needs the support of sufficient models and experimental paradigms [4, 5].

With the rapid development of the information age and the increasing improvement of human living standards, people are increasingly aware of the importance of emotions to their own health and social development [6–9]. Emotion monitoring and recognition is an important part of physiological and psychological medicine. The research on emotion recognition has also been widely applied to real life as follows: in the aspect of auxiliary medical treatment, emotion recognition research can assist patients suffering from depression, autism, and other psychological diseases to formulate effective treatment plans [10, 11].

In recent years, a large number of feature extraction methods of EEG signals have been proposed, but due to the large individual differences, the current research goal is to extract effective features to improve the accuracy of emotion recognition. In the process of signal acquisition, most studies adopted 32- and 64-electrode guide of EEG acquisition. This paper applied the EEG feature extraction and selection into channel emotion recognition, using as few channels as possible to extract effective feature information, to achieve considerable recognition accuracy. The research methods of emotion recognition have many directions. The common research method is to study the recognition and classification of emotions through some physiological characteristics, including facial expressions, behaviors, language, and intonation [12–15]. Although these external physiological characteristics are easy to acquire, their physiological performance may be easily influenced by human consciousness control; artificial control of these physiological characteristics can bias the results of emotion recognition. Therefore, using some physiological signals to identify and classify emotions has become a research hotspot for experts and scholars, such as ECG, pulse, blood pressure, respiration, skin impedance, and other physiological signals. American researchers led their experimental team to prove that physiological signals can identify and classify emotions first [16–19].

The traditional research on emotion recognition is to extract the energy features of EEG signal band or various entropy features to identify an emotion and then use *k*-nearest neighbor and support vector machine classifier to identify emotion [20, 21]. Finally, the recognition effect of each frequency band under several classifiers was compared, and the key frequency domain of the EEG signal was obtained [23]. At the same time, two kinds of emotions were analyzed. The model is studied using binary classification, and the classification recognition rate is improved obviously [24, 25].

The contributions of this paper are concluded as follows:

1. In this paper, the ICA model and feature extraction are innovatively combined to integrate the advantages of both.
2. The existing methods are generally limited to the training of athletes. This paper not only deals with the training of athletes but also studies the selection of athletes.

## 2. Related Work

When people imagine unilateral hand movements, a region of the contralateral cortex becomes active in association with an image called an event in which the amplitude of a specific pitch decreases [26–28]. At the same time, the ipsilateral brain is in a quiet state and the amplitude of related rhythm increases. Relevant theories prove that when people imagine left and right, the rhythm related to it changes in accordance with the above rules. Therefore, this paper uses the above phenomenon and selects the corresponding EEG signal processing method to study the brain imagination signal. The higher the probability of the event, the lower the amplitude of the potential. In a brain-computer interface system based on evoked potentials, the subjects can stimulate the generation of potential and facilitate the extraction of relevant features with a very short training time. It is worth noting that the subjects need to have a high degree of concentration and the stimulation duration is not too long. Bouallegue et al. [29] improved the robot with speech function and added the emotion detection function of speech on the basis of its function. The research team extracted six speech features and some parameters. The correlation feature selection method is used to reduce the dimension of feature vectors, and the radial basis network, Bayesian network, and support vector machine are used to compare the classification accuracy. Campbell et al. [30] used multiple physiological signals (ECG, EMG, respiratory skin electroencephalograph) for feature extraction, relief algorithm for feature selection, and finally J48 decision tree classifier for recognition of four emotions, with an average recognition rate of more than 90%. Raiesdana [31] used a novel music video stimulation to induce the emotions of 32 volunteers and recorded the expression generated by 32 subjects' faces when they watched 40 videos to autonomously evaluate the EEG signals and other physiological signals.

Qazzaz et al. [32] predicted the change of emotion by using Isomap in manifold learning, and the emotional change curve predicted by Isomap was in good agreement with the collected emotion change curve. Zhu et al. [33] used the common space model algorithm to select feature vectors for emotional EEG signals in the DEAP database and divided them into calm and pressure by support vector machine, with an average classification accuracy of 70.84%. New classification models have been used in the study of emotion recognition, and they achieved good classification effect. With the continuous development of brain science and neurology, the researchers began to study emotions and the relationship between specific brain regions, through the connection between them and looking for strong correlation characteristics. In practical applied research, because the aggregation degree of physiological characteristics related to emotion is relatively low, it has a great relationship with people's periodic physical conditions and different people have different emotional EEG patterns. How to obtain a general EEG emotional model through research has become a hot issue that researchers pay attention to. The factors that induce emotion are extremely important in the process of emotion generation and in the study of emotion recognition and classification, and obtaining experimental emotional data is an extremely important part. The commonly used method of research is to use various materials to stimulate the occurrence of emotions and record the EEG signals of other studies. These stimulus materials usually include emotional pictures and audios and videos with emotional colors.

Feature extraction plays an extremely important role in the research of emotion recognition based on EEG signals. Only by selecting the right features that can effectively represent emotion can the following classification research be carried out. Experts have conducted a large number of correlation experiments on feature extraction based on EEG signals, and combining with psychology, physics, and neuroscience, they have identified EEG signatures that can be used to identify emotions. Anem et al. [34] extracted p-QRS-T wave energy features for emotion recognition and classification research and obtained through experiments that such features have a good characterization of emotional states, especially in normal emotions such as optimism and happiness, which play a great role in classification results. Asadur Rahman et al. [35] took advantage of the frequency characteristics of EEG signals and extracted the asymmetry and incoherence of EEG signals as features to identify and classify three types of emotions (anger, fear, and surprise), with an average recognition accuracy of 66.3, selected 6 electrodes from the collected electrodes as the research object, and carried out 5-layer wavelet decomposition on the original signal. The wavelet entropy variance and power of each layer of the 6 electrodes were selected as the classification characteristics, and good classification results were obtained. Amin et al. used the feature extraction scheme of high-order cross analysis to extract the emotional features of EEG signals. Through a comparative experiment between quadratic discriminant *K*-nearest neighbor and support vector machine, emotions were divided into five types: surprise, anger, fear, disgust, and sadness. The highest average recognition rate reached 83.33% with support vector machine. In the process of feature selection, in order to construct a better model, it is necessary to select specific features from a certain number of feature sets. However, in the practical application of pattern recognition, research is faced with features of more dimensions extracted from a large number of data, including features with high correlation and low correlation and features without correlation. The more the number of features, the higher the dimension of the feature vector, the longer the analysis and calculation time, and the more complex the model will be; the recognition rate cannot be guaranteed to be high, so the selection of features is of great significance in the research.

## 3. Key Feature Extraction of the EEG Signal by the ICA Model

### 3.1. Overall Feature Extraction System

In the process of EEG emotion recognition, feature extraction is extremely important and the quality of extracted features directly affects the accuracy of final recognition. The feature extraction methods of EEG mainly include linear time domain, frequency domain, time-frequency direction, nonlinear differential entropy permutation entropy, approximate entropy, and combination entropy. Throughout the experiment, we used EEG signals from the international open data set DEAP, since we use the signal of each guide containing data from 63 s (8064 sampling points) and 60 s and only one is used to stimulate the data according to the experiment; so before the feature extraction of EEG signals, we first need to obtain brain electrical signals to carry out the simple processing.

**Figure 1:**
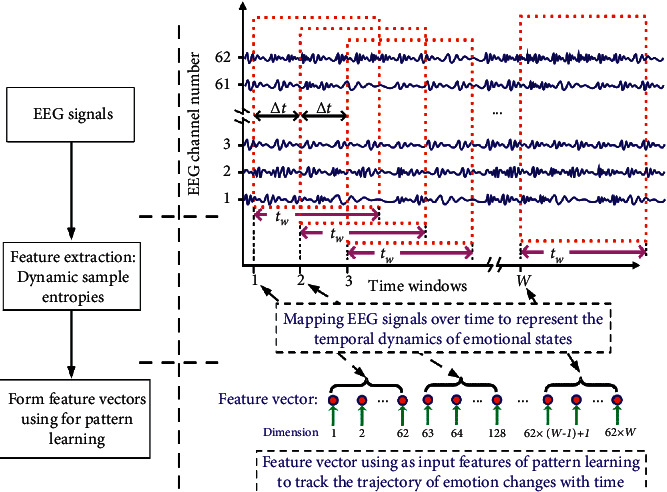
Feature vector extraction method of the EEG signal based on ICA.

Some researchers believed that the data fragments of the first 20 s were collected in an unstable state because the data of the last 40 s would be selected as the research object. ICA is the result of the different frequencies with a cosine function; this function to some extent can express the original signal information, and its essence is to convert the chaotic signal into frequency phase A sinusoidal signal amplitude that has a fixed rule. Because previous studies have shown that EEG emotion recognition can signal the rhythmicity of interest rate, in the process of EEG signal processing, a more classical approach is to obtain the power spectrum of the signal through Fourier transform to calculate the energy value of each frequency band. The whole system of the method is given in Figure 1.

### 3.2. ICA Model

Independent component analysis (ICA) is a signal processing method that attempts to recover independent sources from a group of mixed observations. It is closely related to a method called blind source separation. Shortly after the discovery of the ICA, it was widely used in the processing and analysis of biomedical signals and images. FastICA algorithm is an efficient computing method for ICA estimation. It uses a fixed-point iterative scheme, which has been shown in independent experiments to be 10–100 times faster than ICA's traditional gradient descent method. Another advantage of the FastICA algorithm is that it can also be used to perform projection tracing, thus providing a general data analysis method that can be used both in an exploratory manner and for independent component estimation. Mixed observations *X* are obtained by linear mixing of *K* independent sources *S*, where *A* is called the mixing matrix:

(1) $$\begin{array}{c} \left[\begin{array}{c} x_{1}\left(t\right)\\ x_{2}\left(t\right)\\ \vdots \\ x_{m}\left(t\right) \end{array}\right]=A\left[\begin{array}{c} s_{1}\left(t\right)\\ s_{2}\left(t\right)\\ \vdots \\ s_{k}\left(t\right) \end{array}\right]. \end{array}$$

ICA unmixing first needs to whiten *X* to obtain the unrelated columns [12]as follows:

(2) $$\begin{array}{c} \tilde{X}=ED^{-1/2}E^{T}As=\tilde{A}s. \end{array}$$

FastICA iterative algorithm: after whitening, it is necessary to find an optimal direction *W* to maximize the non-Gaussianity of this direction:

(3) $$\begin{array}{c} J_{G}\left(W\right)={\left[E\left\{G\left(W^{T}X\right)\right\}-E\left\{G\left(V\right)\right\}\right]}^{2}. \end{array}$$

Its derivative under constraints is as follows:

(4) $$\begin{array}{c} \frac{\partial J_{G}\left(W\right)-\beta \left({\left\|W\right\|}^{2}-1\right)}{\partial W}=2E\left\{XG^{\prime }\left(W^{T}X\right)\right\}-2\beta W=0. \end{array}$$

The optimal *w*-union can be obtained by solving the above equations:

(5) $$\begin{array}{c} E\left\{XG^{\prime }\left(W_{0}^{T}X\right)\right\}-\beta W_{0}=0. \end{array}$$

This kind of problem can be solved by Newton's method:

(6) $$\begin{array}{c} x_{n+1}=x_{n}-\frac{f\left(x_{n}\right)}{f^{\prime }\left(x_{n}\right)}. \end{array}$$

So, formula (4) can be formulated as follows:

(7) $$\begin{array}{c} \frac{\partial E\left\{xG^{\prime }\left(w^{T}x\right)\right\}-\beta w}{\partial w}=E\left\{xx^{T}G^{\prime \prime }\left(w^{T}x\right)\right\}-\beta I. \end{array}$$

Therefore, it can be approximated as follows:

(8) $$\begin{array}{c} E\left\{xx^{T}G^{\prime \prime }\left(w^{T}x\right)\right\}\approx E\left\{xx^{T}\right\}E\left\{G^{\prime \prime }\left(w^{T}x\right)\right\}=E\left\{G^{\prime \prime }\left(w^{T}x\right)\right\}I. \end{array}$$

Using Newton's formula [27],we can obtain

(9) $$\begin{array}{c} w_{n+1}=w_{n}-\frac{E\left\{xG^{\prime }\left(w_{n}^{T}x\right)\right\}-\beta w_{n}}{E\left\{G^{\prime \prime }\left(w_{n}^{T}x\right)\right\}-\beta I}. \end{array}$$

The recursive formula is obtained.

(10) $$\begin{array}{c} w_{n+1}=\frac{E\left\{G^{\prime \prime }\left(w_{n}^{T}x\right)\right\}w_{n}-E\left\{xG^{\prime }\left(w_{n}^{T}x\right)\right\}}{E\left\{G^{\prime \prime }\left(w_{n}^{T}x\right)\right\}-\beta },\\ w_{n+1}=E\left\{xG^{\prime }\left(w_{n}^{T}x\right)\right\}-E\left\{{G^{\prime \prime }}^{\left(w_{n}^{T}x\right)}\right\}w_{n}. \end{array}$$

The usual *G*′s are as follows:

(11) $$\begin{array}{c} G_{1}\left(u\right)=\frac{1}{a_{1}}\log\cosh\left(a_{1}u\right),\\ G_{1}^{\prime }\left(u\right)=\tanh\left(a_{1}u\right). \end{array}$$

## 4. Experimental Results and Analysis

### 4.1. Introduction to the Experimental Environment and Data Set

In related studies on EEG emotion recognition, some researchers use self-collected data sets. Because the process of obtaining self-collected data sets is complicated and the cost is high, the data set used in this study is DEAP as an open data set. DEAP is a multimodal emotion data set generated by video clips stimulation. The data set generates 40 channels of physiological signal data per experiment. In the studies related to emotion recognition, only the former is required. The EEG signal of 32 conduction data and the other 8 derivative data including ECG signal and respiratory band signal are not very relevant to emotion recognition, so those will not be described here. Next, the data format of an experimental flow path of acquisition equipment of the DEAP data set will be introduced in detail.

The Biosemi Active Two system was used to collect EEG signals for DEAP data, and prestation software was used to play the video of the emotion generated by the selected stimulus. In order to eliminate the error caused by eye movement in the experimental results, the range of playing the video was 2/3 of the video screen. In addition, the system is relatively compatible with signal reception hardware and is smaller and lighter in weight. In the process of DEAP data set collection, 32 EEG-related electrodes among 128 electrodes were selected for the collection and the signal sampling frequency was 512 Hz. Due to the strong correlation between similar EEG signals, using all 128 electrodes would lead to overlapping of collected signals in this data set. The 32 electrodes are positioned in accordance with international standards and are evenly distributed throughout the brain, containing information from various brain regions.

### 4.2. Experimental Result Analysis

After preprocessing, the labeled EEG data of each subject were divided into a window length of 5 seconds. If the labeled continuous data segment was less than 5 seconds, the labeled EEG data segment of each subject was removed. Then, the labeled EEG data segment of each subject underwent fast-Fourier transform (FFT) in the form of nonoverlapping sliding window to carry out energy calculation. Finally, the average of the energy of data was taken as the subjects of the frequency band energy.

**Figure 2:**
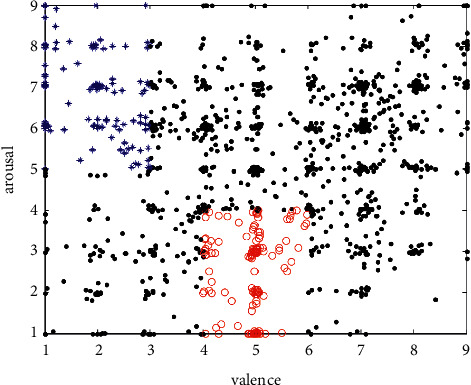
The total number of valid samples taken from 32 people. The abscissa indicates valence, and the ordinate indicates arousal. The black dot indicates worthless samples, the blue asterisk indicates pressure, and the red circle indicates calm.

The database collected 32 volunteers' EEG signals stimulated by 40 segments of the material. There were 40∗32 = 1280 emotion samples in total, among which 270 samples met the classification criteria.  Figure 2 shows the samples extracted from the data of volunteer No. 1.

**Figure 3:**
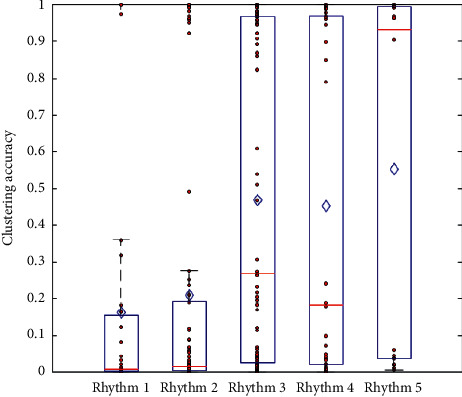
Cluster analysis results of different level features.

**Figure 4:**
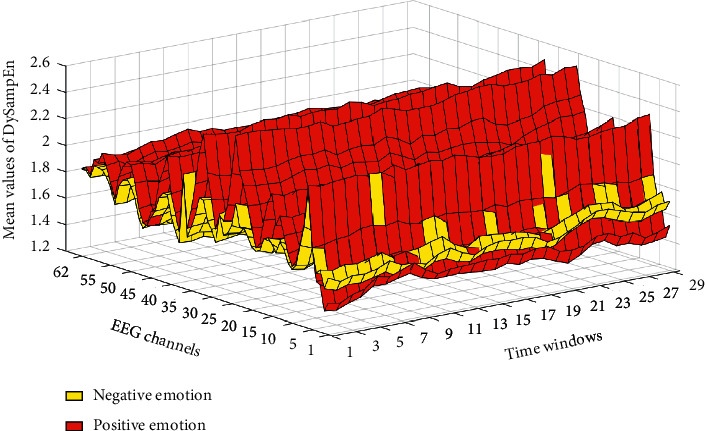
Distribution of EEG characteristics under different states.

With the development, the innovation hot spots in the payment field are further evolving from payment mode innovation to payment medium innovation. New technologies such as code payment, biometric payment, and NFC are emerging, and their application scope is gradually expanding. According to the iResearch data statistics, in 2017, by virtue of the intelligent transformation of traditional POS machines, bank card orders accounted for 19.4% of the payment market size, Internet payment accounted for 16.8%, and mobile payment accounted for 63.7% which has become the absolute leader in new payment formats. In addition, the results of the frequency method of Beta rhythm mode based on cluster analysis are shown in Figure 3. It can also be seen that the frequency values of each group show an overall upward trend with the increase in game level, while the results of the frequency method of Beta rhythm mode based on linear discriminant analysis are shown in Figure 4. The frequency values of all game level groups showed an upward trend with the increase in game level. Finally, the relative energy of Beta3 in level 5 group was significantly lower than that in level 1 to level 3 groups based on Beta rhythm analysis using the traditional statistical analysis method. The frequency method of the Beta rhythm mode proposed in this paper has good practical effect and robustness. The results also indicate that the Beta rhythm mode can describe the influence of game level on the attention-related brain function of subjects. This analysis method can understand the significance of brain electrical rhythm and the influence of AVG on brain plasticity from a new perspective. It has good application potential and prospects.

**Figure 5:**
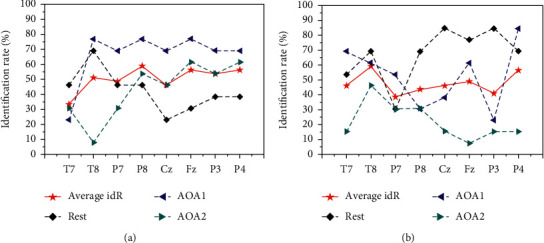
Results of EEG feature recognition of each channel based on (a) ICA and (b) SVM.

Here, the features mentioned above in the time domain are extracted and the recognition results of single feature and combination feature are compared. It is then compared with time-frequency-based features separately. All the comparisons were performed on all 32 subjects who watched 40 movie clips, each of which generated data from all 32 channels (electrodes). Next, we verify the validity of the feature extraction algorithm using points, and the comparison of results is shown in Figure 5.

**Figure 6:**
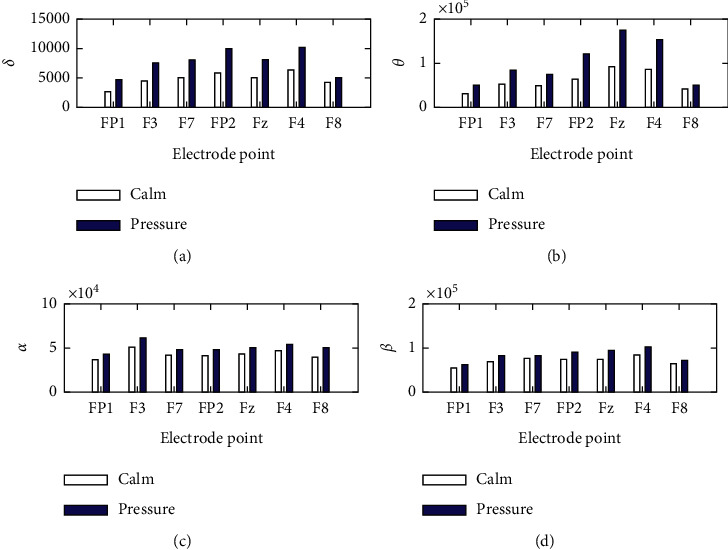
Comparison of features in each frequency band. (a) Rhythm 1. (b) Rhythm 2. (c) Rhythm 3. (d) Rhythm 4.

In order to evaluate the difference in the performance of the EEG measures corresponding to each channel for attention recognition of auditory targets, based on the SVM auditory attention classifier, the experiment further discussed the EEG features corresponding to each channel as the input feature vector.  Figure 6 shows the recognition results of auditory target attention achieved by the ICA-based auditory attention classifier with EEG approximate entropy and composite multiscale entropy of each channel as input feature vectors.

**Figure 7:**
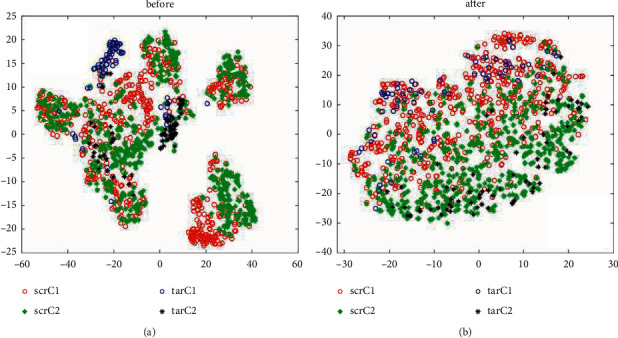
Selection results before and after feature extraction. (a) Classification results without ICA extraction. (b) Classification results after extracted with ICA.

As shown in Figure 7, in the study of emotional EEG signals, electrical signals are generally divided into the time domain, frequency domain and time-frequency characteristic of three case studies, and emotion recognition research association is the largest frequency-domain feature. Frequency band energy is generally regarded as a classification feature, and the energy value of a person under pressure is generally greater than that of a person under calm emotion. Medically speaking, when a person is under pressure, the brain is active and tense, the brain processing intensity of information increases, and the interaction between various nerves becomes more frequent. Therefore, under pressure, the frequency band energy of the EEG signal increases.

**Figure 8:**
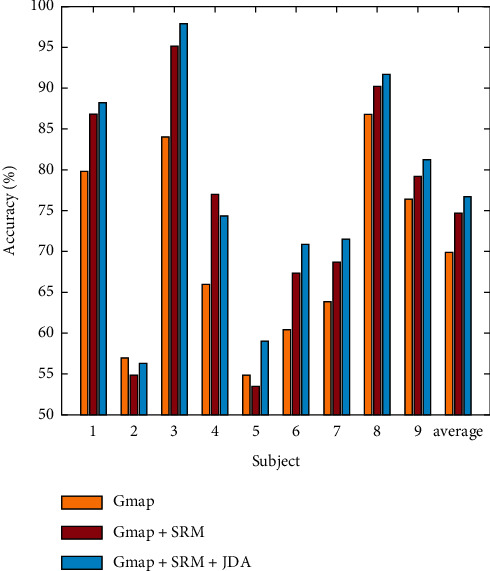
The selection accuracy of this method under different experimental conditions.

The effectiveness of the migration method is further verified, and the effect of the migration method is vividly demonstrated. The feature distribution before and after the migration is visualized in this small pair. Here, T-SNE technology is used for feature dimension reduction and data visualization of extracted features. T-SNE can reduce high-dimensional data to 2-3 dimensions, and the features after dimension reduction can vividly show the distribution of features.  Figure 8 shows the feature distribution of the lowest and highest subjects (subjects 2 and 5) before and after the migration of athletes' selection accuracy. The red and blue circles in the feature graph represent the first-class features of the source and target domain objects, and the green and the black star represent the second-class features of the source and target domain objects. The feature distribution of each category is more similar, that is, red and blue clusters together with circles, green, and black clusters achieve the purpose of feature migration.

Figure 8 shows each component in the objective function of the algorithm: the influence of manifold feature item Gmap, structured risk item SMR, and joint distribution alignment item JDA on the test accuracy of each subject in the two data sets. It can be seen that due to the large individual difference of data set 1, the joint distribution alignment item has a significant impact on the algorithm performance. It can be seen that after the addition of SRM, except for subject 2 and subject 5, the effect of other subjects is significantly improved. SRM is used to avoid the error risk of the classifier, indicating that only migration is not enough, and the migration combined with the classifier may improve the accuracy to a certain extent.

## 5. Conclusions

This paper mainly studies feature extraction, channel selection, and classification model selection of emotion recognition based on EEG. At present, research studies on emotion recognition based on EEG signal have low recognition accuracy. In this paper, the method of feature extraction is improved and the ICA model with excellent performance in data mining is selected for classification. Compared with some existing studies, the recognition accuracy of emotion recognition by the feature extraction method proposed in this paper has been improved to some extent.

Emotion recognition is studied in this paper, based on EEG signals, comparing only the electrical characteristics of the time domain and the time-frequency combination and not the electrical characteristics and other properties of the mixed emotion recognition results; in the process of emotion recognition, due to the individual difference being greater, the identification results of this paper are the emotion recognition results of each people, respectively. Therefore, in subsequent studies, we will explore the emotion recognition results of the combination of EEG signal features and other physiological signal features. For problems with large individual differences, we will try to use a deep learning algorithm to carry out relevant studies.

## Data Availability

The data used to support the findings of this study are available from the corresponding author upon request.
